# A Trans-omics Mathematical Analysis Reveals Novel Functions of the Ornithine Metabolic Pathway in Cancer Stem Cells

**DOI:** 10.1038/srep20726

**Published:** 2016-02-11

**Authors:** Jun Koseki, Hidetoshi Matsui, Masamitsu Konno, Naohiro Nishida, Koichi Kawamoto, Yoshihiro Kano, Masaki Mori, Yuichiro Doki, Hideshi Ishii

**Affiliations:** 1 Department of Cancer Profiling Discovery, Graduate School of Medicine, Osaka University, Osaka 565-0871, Japan; 2 Faculty of Mathematics, Kyushu University, Fukuoka, 819-0395, Japan; 3 Department of Frontier Science for Cancer and Chemotherapy, Graduate School of Medicine, Osaka University, Osaka 565-0871, Japan; 4 Department of Gastroenterological Surgery, Graduate School of Medicine, Osaka University, Osaka 565-0871, Japan

## Abstract

Bioinformatics and computational modelling are expected to offer innovative approaches in human medical science. In the present study, we performed computational analyses and made predictions using transcriptome and metabolome datasets obtained from fluorescence-based visualisations of chemotherapy-resistant cancer stem cells (CSCs) in the human oesophagus. This approach revealed an uncharacterized role for the ornithine metabolic pathway in the survival of chemotherapy-resistant CSCs. The present study fastens this rationale for further characterisation that may lead to the discovery of innovative drugs against robust CSCs.

Recently, many transcriptome and metabolome analyses have been reported. Transcriptome analyses have been extensively used to investigate gene expression in individual cells. On the other hand, metabolome analyses have been used to identify changes in the biochemical behaviour of metabolites, such as amino acids, fatty acids and other organic substances. Initial efforts have been separately focused on the two analyses, mainly because of a lack of appropriate models or methods for analysis. However, it is clear that the strong correlation between the resulting datasets would be useful for predicting the biophysical and biochemical behaviours of cells. Accordingly, the development of mathematical models of the relationship between transcriptome and metabolome analyses is highly desirable.

In medical science, recent studies have suggested that the subpopulations of tumour-initiating cells, or cancer stem cells (CSCs), are responsible for the heterogeneity of tumours. This heterogeneity is involved in their refractoriness to chemoradiation therapy as well as their subsequent relapse^1^. In an analogy to somatic stem cells, CSCs reportedly survive in hypoxic areas. Hypoxic glycolysis refers to the production of lactate and to a lesser extent to the contribution of oxidative phosphorylation and the additional relevance of reactive oxygen species production in mitochondria^2^. Furthermore, recent studies have indicated that stimulation for the induction of the epithelial-to-mesenchymal transition results in the activation of the CSC property^3^,^4^, suggesting that the elucidation of transcriptome and metabolome linkages may allow the precise prediction of the biological behaviours of CSCs.

We recently reported that a fluorescence-based vector with a degron motif originating in ornithine decarboxylase (ODC) was useful for the visualisation of CSCs^5^,^6^,^7^,^8^,^9^. In these reports, the relationship between proteasome activity and ODC in oesophageal, cervical, colorectal and bone cancer was investigated. Previous studies by others and ourselves showed that although proteasome activity was not associated with the intensity of green fluorescent protein fused by the degron motif, cells sorted by fluorescence indeed showed high tumourigenicity and therapy resistance. These properties correspond to those of CSCs^5^,^6^,^7^,^8^,^9^, indicating that the degron vector system was useful for tracing CSCs and suggesting that cancer stemness may reflect intracellular natures other than only proteasome activity. We accordingly studied the combination of transcriptome and metabolome in degron-positive CSCs and degron–negative non-CSCs. To this end, we developed novel technologies that combine the transcriptome and metabolome for identifying the events occurring in CSCs that support cell survival during exposure to therapy. We performed a bioinformatic and computational modelling study to link the transcriptome and metabolome for CSCs and ‘differentiated cancer cells’ (non-CSCs) and then compared these two results (Fig. 1). This approach allowed the identification of a novel function of the ornithine metabolic pathway as an important feature in the survival of chemotherapy-resistant CSCs.

## Results

**Figure 2:**
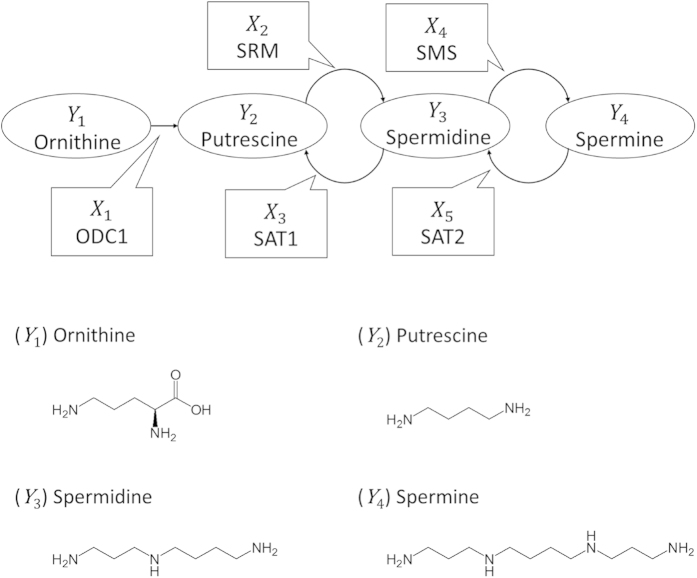
The relationship between our simple reaction model for polyamine metabolism and associated enzymes, (*X*_1_) ODC1, (*X*_2_) SRM, (*X*_3_) SAT1, (*X*_4_) SMS and (*X*_5_) SAT2, as well as the structural formulas of (*Y*_1_) ornithine, (*Y*_2_) putrescine, (*Y*_3_) spermidine and (*Y*_4_) spermine. ODC, ornithine decarboxylase; SAT2, spermine *N*1-acetyltransferase 2; SMS, spermine synthase.

To identify the events occurring in CSCs in response to therapy, we performed a bioinformatic study of the metabolites and enzymes involved in polyamine metabolism. In our analysis, three independent moieties involved in polyamine metabolism were considered, as shown in Fig. 2. The first moiety focused attention on only the reactions of inflow to and outflow from putrescine (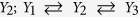). The second moiety focused on only the compounds around spermidine (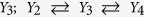). The final moiety focused on the interconversion of spermidine and spermine (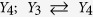). We defined these three reaction moieties as the putrescine, spermidine and spermine moieties, respectively. Our analysis focused on the variability of the reaction coefficients over time after exposure to 5-fluorouracil (5-FU) or cisplatin (CDDP). Table 1 shows the coefficients obtained from fitting each reaction moiety in cells displaying high concentrations of ZsGreen-cODC (Zs + ; CSCs) or low concentrations of ZsGreen-cODC (Zs−; non-CSCs), as well as cross-validation (CV) errors. We considered that the CV errors that resulted from our parameter fitting were sufficiently small to be useful for qualitative understanding.

We first investigated transcription and metabolism involved in the polyamine metabolism pathway at the zero time point (*T*_0_). As shown in the supplemental Fig. 1, there was no difference between CSCs and non-CSCs in the amount of enzyme transcription. The metabolome analysis indicated that CSCs have higher putrescine and spermidine contents than non-CSCs, downstream cell populations, whereas ornithine was lower in non-CSCs, showing that the flux of polyamine metabolism plays a role in biosynthesis in CSCs, compared with non-CSCs, at *T*_0_.

**Figure 3:**
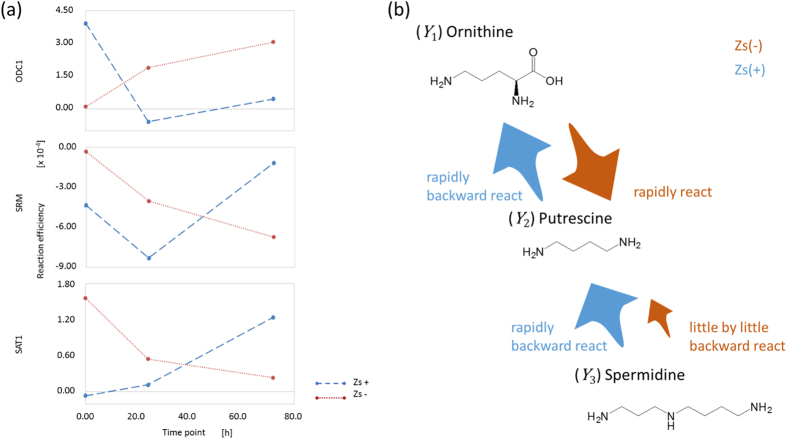
(**a**) The change over time of the coefficients of variability for ODC1, SRM and SAT1 after exposure of Zs + and Zs− cells to anti-tumour agents and (**b**) the difference in reaction flow via these metabolites for the putrescine moiety. ODC, ornithine decarboxylase; SAT2, spermine *N*1-acetyltransferase 2.

**Figure 4:**
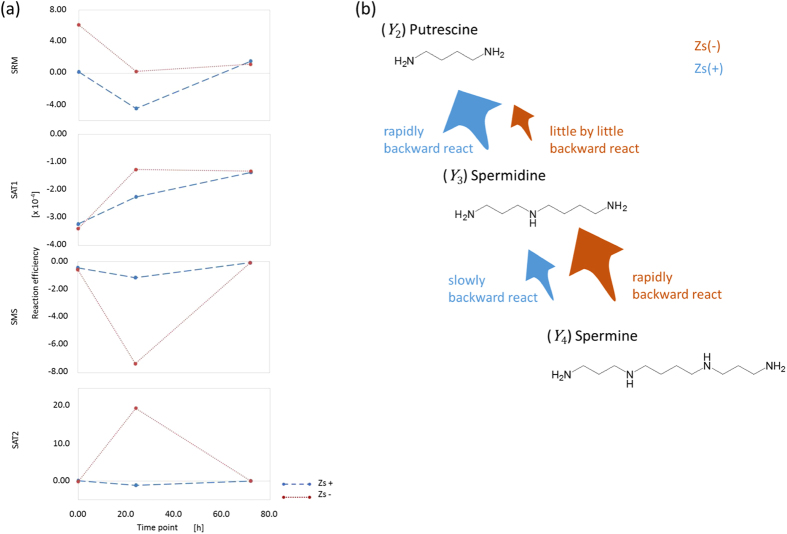
(**a**) The change over time of the coefficients of variability for SRM, SAT2, SAT1 and SMS after exposure of Zs + and Zs− cells to anti-tumour agents and (**b**) the difference in reaction flow via these metabolites for the spermidine moiety. SAT2, spermine *N*1-acetyltransferase 2; SMS, spermine synthase.

**Figure 5:**
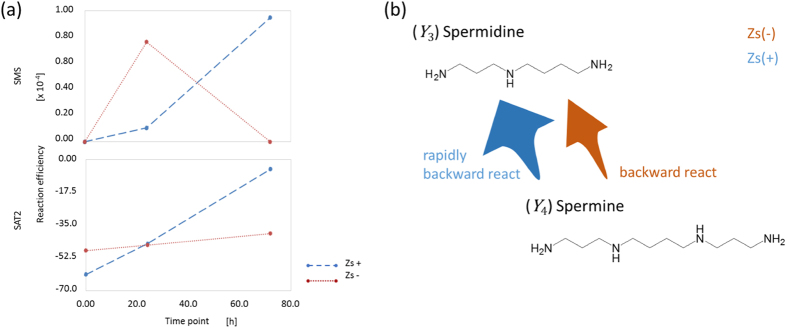
(**a**) The change over time of the coefficients of variability for SMS and SAT2 after exposure of Zs + and Zs− cells to anti-tumour agents and (**b**) the difference of reaction flow via these metabolites for the spermine moiety. SAT2, spermine *N*1-acetyltransferase 2; SMS, spermine synthase.

We show the changes in the coefficients over time as a line graph in Figs 3, 4, 5 (blue dashed line: Zs + , brown dotted line: Zs−), for the putrescine, spermidine and spermine moieties, respectively. These lines do not show interpolation between the time points. Positive values in these plots correspond to acceleration of the forward enzymatic reaction and negative values to that of the backward reaction.

In the putrescine moiety of non-CSCs (Zs−), exposure to an anti-tumour agent accelerates the reaction from 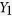 to 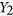. Despite increasing the concentration of 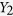, the reaction between 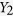 and 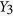 leads to a further increase in the concentration of 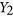 over time. For CSCs, the change in the ODC coefficients over time means that exposure to an anti-tumour agent led to the sudden acceleration of the backward reaction, although some recovery could be seen 72 h later. In addition, the reaction from 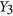 to 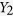 is promoted. This promotion means that CSCs struggled to maintain the concentration of 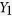 during the rapid reverse reaction, but that non-CSCs became conducive to conversion of 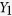 into 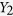.

With respect to the spermidine moiety, we propose that the changes in the coefficients over time show similar tendencies in CSCs and non-CSCs. In the reaction between 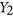 and 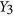, non-CSCs inactivated the reaction, whereas CSCs slowed it after promoting the reverse reaction. This behaviour means that the exposure to the anti-tumour agent maintains the amount of 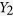, with a low reverse reaction rate from 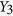 to 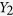 in both cells. In contrast, in the 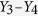 reaction, we found a very interesting tendency upon exposure to the anti-tumour agent. The conversion changes rapidly to the reverse reaction (from 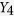 to 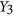) in differentiated cancer cells, whereas the changes show little response in CSCs. In other words, within the limitation of the spermidine moiety, although the polyamine metabolism reaction was controlled to maintain the concentration of putrescine in non-CSCs, the reaction toward putrescine was promoted in CSCs. Finally, the coefficient order of spermidine/spermine *N*1-acetyltransferase 2 (SAT2) is different from that of spermine synthase (SMS) at the spermine moiety. Our analysis may capture the feature of promoting the reaction from 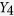 to 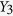 in both CSCs and non-CSCs. Additional information about the coefficient order is presented in the Supplemental Fig. 2. We could find out that there are some enzyme governed and not contributed the direction of pathway flow in each reaction moiety. For example, in Spermidine moiety, SMS and SAT2 make a larger contribution to decision of direction of pathway flow. Meanwhile, SRM and SAT1 have less contribution.

## Discussion

**Figure 6:**
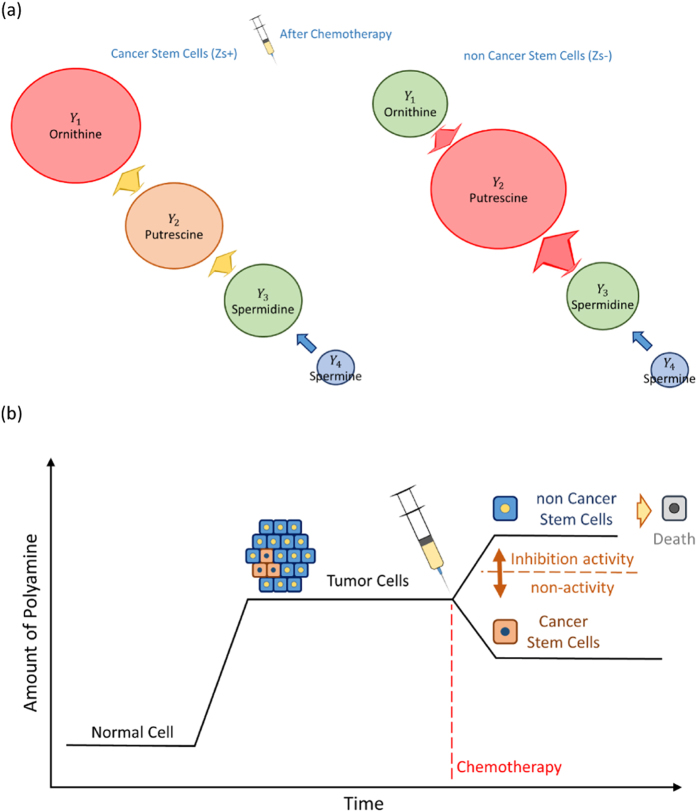
(**a**) A graphical image of reaction responses after exposure to anti-tumour agents in cancer stem cells (left) and non cancer stem cells (right). (**b**) A schematic diagram of the relationship between the total amount of polyamine and cancer cell survivability.

Considering a future application of reaction pathways whose detail of inflow and outflow are unknown, we have divided the reaction pathways into independent moieties. In our analysis, however, we should not compare the absolute values of coefficients between reaction moieties, because the coefficients of each moiety are estimated independently. Thus, in our analysis, it is essential to understand direction of pathway flow in each reaction moiety. In view of the qualitative changes in the coefficients of each reaction moiety over time, the inflow to putrescine in non-CSCs after exposure to anti-tumour agent stands out, as its reaction rate slowed with time. In contrast, it appears that the polyamine reaction is controlled in such a way as to increase ornithine to a certain level in CSCs, although the general reaction variation is small compared with that of non-CSCs, as shown in Fig. 6a.

Cancer cells have higher levels of ornithine-derived polyamines than normal cells, and polyamines contribute to cell growth, survival and proliferation^10^. Thus, polyamine metabolism is strictly controlled in cancer cells under physiological conditions^11^. The present study showed that CSCs reduce their polyamine level when exposed to 5-FU or CDDP. This reduction leads to the deceleration of the cell cycle. In contrast, non-CSCs increase their polyamine level when exposed to 5-FU or CDDP, leading to an acceleration of the cell cycle. 5-FU and CDDP are effective against cells with a high multiplication rate. It thus appears that a reduction in polyamines is an important strategy of CSCs for resisting the effects of 5-FU or CDDP (Fig. 6b). Moreover, previous reports have suggested that polyamine catabolic enzymes such as SAT1, SAT2, *N*1-acetylpolyamine oxidase and spermine oxidase (SMO) share high similarity in overall structure with the histone demethylase LSD1. The amino acid sequence of SMO, in particular, shares over 60% similarity with that of LSD1^12^. This similarity suggests that polyamines inhibit LSD1, which act as an epigenetic regulator of histone demethylase^13^. The histone methylation level in gene promoter regions regulates the level of gene expression. For example, H3K4 me1 (monomethylated H3K4) marks gene enhancers, whereas H3K4 me3 (trimethylated H3K4) associates with active promoters of gene expression^14^. Many studies have indicated that LSD1 function has an important role in cancer. Cancer cells that highly express LSD1 contribute to increasing risk of cancer recurrence, suggesting that LSD1 promotes cancer survival^15^. For this reason, it is thought that inhibition of LSD1 is a key phenomenon in the reactivation of silenced tumour suppressor genes in cancer cells^16^. In our analysis, CSCs showed decreased polyamine levels during chemotherapy and resisted this therapy. These results suggest that polyamines inhibit LSD1 enzyme activity and change gene expression levels to influence cell survival. In this study, we showed that CSCs reduce polyamine levels to resist chemotherapy. In contrast, polyamines in non-CSCs are maintained at a high level. This high polyamine level may act to inhibit LSD1 activity and induce epigenetic changes that increase cell survival, as suggested in previous reports. The present study shows that a mathematical model connecting transcriptome to metabolome analysis is a powerful approach to understand the multiple features of biological pathways in the fields of cancer research and medical science.

## Methods

### Cell culture and sorting

We purchased the human oesophageal cancer cell lines TE-4 and TE-8 from the Japanese Collection of Research Bioresources Cell Bank (Ibaraki, Japan). These cells were cultured in Dulbecco’s modified Eagle’s medium (DMEM; Sigma-Aldrich, St. Louis, MO, USA) supplemented with 10% fetal bovine serum (FBS; Hyclone, Logan, UT, USA) and penicillin-streptomycin (Sigma-Aldrich) in an atmosphere of 5% CO_2_ at 37 °C. We used the retroviral vector pQCXIN-ZsGreen-cODC, which encodes the ZsGreen-cODC fluorescent fusion protein^6^,^7^. The Platinum-A Retroviral Packaging Cell Line (Plat-A) was adopted. Plat-A was optimized to produce high retroviral titers. We purchased the cells from Cell Biolabs (San Diego, CA, USA). These cells were cultured in DMEM supplemented with 10% FBS, 100 U/ml penicillin (Life Technologies, Gaithersburg, MD, USA), 1 μg/ml promycin (Sigma-Aldrich) and 10 μg/ml blasticidin (Sigma-Aldrich). To generate retroviruses, we transfected Plat-A cells with the retroviral vector described above using the FuGENE6 transfection reagent (Promega Corp., Madison, WI, USA). At 1 day after transfection, the medium was changed. One additional day later, the supernatant containing the retroviruses was collected. To induce cancer cell formation, we added this supernatant and 6 mg/ml polybrene (Sigma-Aldrich) to DMEM containing the cultured cancer cells. Cells with high ZsGreen-cODC (Zs + ) expression and low ZsGreen-cODC (Zs−) expression were separated after two rounds of FACS and defined as CSCs and non-CSCs, respectively. We washed these cells with phosphate-buffered saline, trypsinized them by adding 0.25% trypsin–ethylenediaminetetraacetic acid (Life Technologies) to the medium and sorted them with a BD FACSAria II cell sorter system (Becton-Dickinson, Franklin Lakes, NJ, USA).

### Metabolome analysis

We performed non-targeted metabolome analysis using dishes of cultured cells (10^6^ cells/sample). Targeted cells were first washed twice with 5% mannitol solution and treated with 800 μl of methanol. The extracted cells were then treated with 550 μl of internal standards (H3304-1002, Human Metabolome Technologies, Inc., Tsuruoka, Japan) in Milli-Q water. After they were left to rest for 30 s, they were centrifuged at 2,300 *g* for 5 min at 4 °C. Additionally, to remove extra proteins, 800 μl of the upper aqueous layer was centrifugally filtered with a Millipore 5 kDa cutoff filter at 9,100 *g* for120 min at 4 °C. To obtain peak information, including *m*/*z* and migration time (MT) from the CE-TOFMS measurement, as well as the peak areas, we recorded the peaks using the automatic integration software package MasterHand (Keio University, Tsuruoka, Japan). After removal of the peaks corresponding to isotopomers, adduct ions and some product ions of known metabolites, the remaining peaks derived from putative metabolites were identified via the HMT metabolite database based on their MT and *m*/*z* values determined by MS. We set the tolerance ranges for peak annotation at ± 0.5 min for MT and ± 10 parts per million for *m*/*z*. The peak areas were normalized against the areas of the internal standard. Furthermore, the relative areas were renormalized by the amount of target sample. We performed hierarchical cluster analyses and principal component analyses using the in-house software packages PeakStat and SampleStat, respectively.

### Microarray analysis

The extracted total RNA (500 ng) was labelled with cyanine-3 using the Low Input Quick Amp Labeling Kit (Agilent) after checking to ensure sufficient quality for microRNA microarray experiments. The cRNA yield and dye incorporation were checked using a NANODrop ND-2000 Spectrophotometer. These labelled RNAs were hybridized with the Agilent Mouse GE 8 × 60 K Microarray in a rotating Agilent hybridisation oven for 17 h at 65 °C. After the hybridisation, the microarrays were washed at room temperature for 1 min with GE Wash Buffer 1 (Agilent, Tokyo, Japan), and then washed for 1 min with GE Wash buffer 2 (Agilent) at 37 °C. Finally, they were dried immediately by brief centrifugation. The fluorescence signals were determined using an Agilent DNA Microarray Scanner (G2565CA) after stringent washes with GE Wash Buffer 1 and 2 (Agilent) for 1 min each. The fluorescence signals were analysed with Feature Extraction Software 10.10 (Agilent).

### Mathematical modelling

Suppose we have 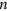 sets of observations 
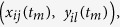
 where 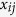 are enzymes, 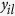 are metabolites,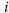 is an index for each subject and 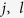 are indices of enzymes and metabolites, respectively. Furthermore, they are repeatedly measured at different time points 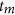 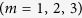, where 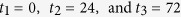. An illustration of the relationship between metabolites and enzymes (transcripts) involved in polyamine metabolism is shown in Fig. 2. In this study, we adopted a simple reaction model with only five enzymes: (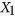), ODC1; (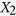), spermidine synthase; (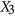), SAT1; (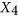), SMS and (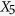), SAT2 and four metabolites: (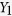), ornithine; (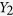), putrescine; (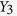), spermidine and (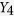), spermine. When we focus on putrescine, the linear model that represents the relationship between enzymes and metabolites has the following form:


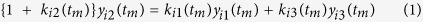


for 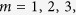 where 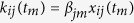 with reaction efficiencies 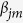 and indices for enzymes 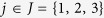. The problem is to estimate 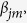 which correspond to the coefficient parameters in regression models.

We consider estimating parameters using the penalized least-squares method^17^; that is, minimizing the following penalized squared criterion with respect to 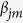:


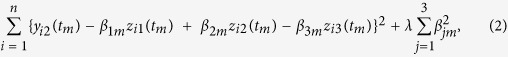


where 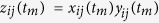 and 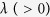 is a regularisation parameter that controls the degree of penalization. Advantages of the penalized least squares method are that it provides more stable estimators than the ordinary least squares method and that it can construct statistical models applicable to the prediction of data to be obtained in the future. Then, we have estimators of coefficient vectors 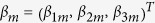 (where 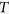 denotes the transpose of a matrix or a vector) as


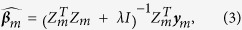


where 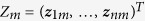 with 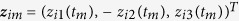, 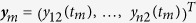, and 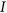 denotes an identity matrix.

Because the estimated model is strongly affected by the value of the regularisation parameter 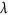, we selected its value using leave-one-out CV^18^. The value of 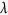 is selected as follows. First, we assign a preliminary candidate value to 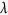. Next, we obtain the estimator of the coefficient parameter 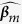 and thus obtain a value for the CV. We repeat this sequence for different values of 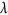 and then obtain the corresponding values of the CV. Finally, we select the 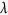 that minimizes the CV and then adopt the corresponding model as the optimal one. For details of such tuning parameter selection, see Green and Silverman^19^. In the above process of CV, it is expected that some parameters influenced by experimental error are excluded.

These coefficient parameters are fitted to our metabolome and transcriptome analysis at the points representing 0, 24 and 72 h after exposure to the anti-tumour agent 5-FU or CDDP. In our analysis, the data of 5-FU and CDDP are treated as equivalent sampling points to fit the best parameters for post-exposure points. Given that the one metabolome and two transcriptome analyses were performed with each anti-tumour agent at each time point, we actually used four sets of experimental data. Under ordinary circumstances, we might have to choose the varying-coefficient model of Hastie and Tibshirani^20^, because the above regression model uses only the data for one time point each and does not leverage the data for the other time points. However, experimental data could be obtained at only a limited number of time points owing to the high cost. Thus, using this small amount of time point data might lead to an incorrect result with the varying-coefficient model, because the parameters have high flexibility. The simplicity of the reaction model of polyamine metabolism shown in Fig. 2 might also increase the possibility of obtaining an erroneous answer. In fact, we found that the error value using the leave-one-out CV was larger than the error for a linear model. For these reasons, we selected the regression model. If we focus on other metabolites, we construct the model in a similar way by reference to Fig. 2.

## Additional Information

**How to cite this article**: Koseki, J. *et al.* A Trans-Omics Mathematical Analysis Reveals Novel Functions of the Ornithine Metabolic Pathway in Cancer Stem Cells. *Sci. Rep.* **6**, 20726; doi: 10.1038/srep20726 (2016).

## Supplementary Material

Supplementary Information

## Figures and Tables

**Figure 1:**
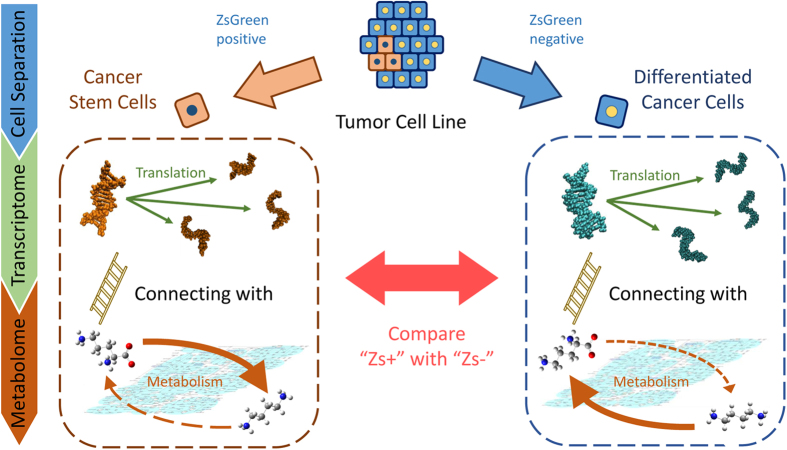
Schematic illustration of our analysis linking transcriptome and metabolome for cancer stem cells and differentiated cancer cells (non cancer stem cells).

**Table 1:** The variability of coefficients for each reaction moiety over time after exposure of Zs + cells (cancer stem calls) and Zs− cells (non cancer stem cells) to anti-tumour agents.

	Time point (h)			
	0	24	72	CV error
Reaction moiety				
Putrescine				
Zs+				
ODC1	3.93E-04	−5.87E−05	4.53E-05	
SRM	−4.33E−04	−8.29E−04	−1.16E−04	1.67E-09
SAT1	−6.59E−06	1.16E-05	1.24E-04	
Zs−				
ODC1	1.00E-05	1.89E-04	3.07E-04	
SRM	−3.22E−05	−4.02E−04	−6.73E−04	3.78E-11
SAT1	1.56E-04	5.47E-05	2.35E-05	
Spermidine				
Zs+				
SRM	1.69E-06	−4.44E−05	1.53E-05	
SAT2	1.15E-05	−1.12E−04	3.67E-07	8.84E-13
SAT1	−3.23E−04	−2.25E−04	−1.37E−04	
SMS	−4.51E−05	−1.16E−04	−8.62E−06	
Zs−				
SRM	6.12E-05	2.48E-06	1.15E-05	1.67E-12
SAT2	−1.03E−05	1.97E-03	1.12E-06	
SAT1	−3.39E−04	−1.27E−04	−1.33E−04	
SMS	−5.85E−05	−7.35E−04	−7.76E−06	
Spermine				
Zs+				
SMS	1.95E-09	1.07E-05	9.49E-05	8.03E-15
SAT2	−6.13E−03	−4.50E−03	−5.04E−04	
Zs−				
SMS	5.13E-09	7.63E-05	6.43E-10	2.79E-15
SAT2	−4.85E−03	−4.57E−03	−3.95E−03	

CV errors are shown for each cell.

CV, cross-validation; ODC, ornithine decarboxylase; SAT2, spermine *N*1-acetyltransferase 2; SMS, spermine synthase.

## References

[b1] Reya T. Morrison S. J., Clarke M. F. & Weissman I. L. Stem cells, cancer, and cancer stem cells. Nature 414, 105–111 (2001). 11689955 10.1038/35102167

[b2] Diehn M. *et al.* Association of reactive oxygen species levels and radioresistance in cancer stem cells. Nature 458, 780–783 (2009). 19194462 10.1038/nature07733 PMC2778612

[b3] Mani S. A. *et al.* The epithelial-mesenchymal transition generates cells with properties of stem cells. Cell 16, 704–715 (2008). 18485877 10.1016/j.cell.2008.03.027 PMC2728032

[b4] AI-Hajj M., Wicha M. S., Benito-Hernandez A., Morrison S. J. & Clarke M. F. Prospective identification of tumorigenic breast cancer cells. Proc. Natl. Am. Soc. 100, 3983–3988 (2002). 10.1073/pnas.0530291100 PMC153034 12629218

[b5] Kano Y. *et al.* Novel drug discovery system for cancer stem cells in human squamous cell carcinoma of the esophagus. Oncol. Rep. 31, 1133–1138 (2014). 24378718 10.3892/or.2013.2952

[b6] Tamari K. *et al.* Identification of chemoradiation-resistant osteosarcoma stem cells using an imaging system for proteasome activity. Int. J. Oncol. 45, 2349–2345 (2014). 25269626 10.3892/ijo.2014.2671

[b7] Hayashi K. *et al.* Visualization and characterization of cancer stem-like cells in cervical cancer. Int. J. Oncol. 45, 2468–2474 (2014). 25269542 10.3892/ijo.2014.2670

[b8] Vlashi E. *et al.* Metabolic state of glioma stem cells and nontumorigenic cells. Proc. Natl. Am. Soc. 108, 16062–16067 (2011). 10.1073/pnas.1106704108 PMC3179043 21900605

[b9] Adikrisna R. *et al.* Identification of Pancreatic Cancer Ste Cells and Selective Toxicity of Chemotherapeutic Agents. Gastroenterology. 143, 234–245 (2012). 22510202 10.1053/j.gastro.2012.03.054

[b10] Agostinelli E. *et al.* Polyamines: fundamental characters in chemistry and biology. Amino Acids 38, 393–403 (2010). 20013011 10.1007/s00726-009-0396-7

[b11] Ruiz-Pérez M. V., Medina M. Á., Urdiales J. L., Keinänen T. A. & Sánchez-Jiménez F. Polyamine metabolism is sensitive to glycolysis inhibition in human neuroblastoma cells. J Biol Chem. 290, 6106–6119 (2015). 25593318 10.1074/jbc.M114.619197 PMC4358251

[b12] Yi H., Marton L. J., Woster P. M. & Casero R. A. Polyamine analogues targeting epigenetic gene regulation. Essays Biochem. 46, 95–110 (2009). 20095972 10.1042/bse0460007 PMC3564236

[b13] Shi Y. *et al.* Histone demethylation mediated by the nuclear amine oxidase homolog LSD1. Cell. 119, 941–953 (2004). 15620353 10.1016/j.cell.2004.12.012

[b14] Baylin S. B. & Ohm J. E. Epigenetic gene silencing in cancer: a mechanism for early oncogenic pathway addiction? Nat. Rev. Cancer. 6, 107–116 (2006). 16491070 10.1038/nrc1799

[b15] Kahl P. *et al.* Androgen receptor coactivators lysine-specific histone demethylase 1 and four and a half LIM domain protein 2 predict risk of prostate cancer recurrence. Cancer Res. 66, 11341–11347 (2006). 17145880 10.1158/0008-5472.CAN-06-1570

[b16] Murray-Stewart T. *et al.* Polyamine analogue inhibition of lysine-specific demethylase 1 in human acute myeloid leukaemia cell lines. Proc. Am. Assoc. Cancer Res. 49, 2605 (2008).

[b17] Hoerl A. & Kennard R. Ridge regression: Biased estimation for nonorthogonal problems. Technometrics 12, 55–67 (1970).

[b18] Stone M. Cross-validatory choice and assessment of statistical predictions. Journal of the Royal Statistical Society. Series B 36, 111–147 (1974).

[b19] Green P. J. & Silverman B. W. Nonparametric regression and generalized linear models: a roughness penalty approach. London: Chapman & Hall/CRC (1994).

[b20] Hastie T. & Tibshirani R. Varying-coefficient models. Journal of the Royal Statistical Society, Series B 55, 757–796 (1993).

