# Supplementary material for: A Trans-omics Mathematical Analysis Reveals Novel Functions of the Ornithine Metabolic Pathway in Cancer Stem Cells
Source: Sci Rep. 2016 Feb 11;6:20726. doi: 10.1038/srep20726 (PMC4749963; doi:10.1038/srep20726)
Supplement: Supplementary Information [file srep20726-s1.doc]

A Trans-omics Mathematical Analysis Reveals Novel Functions of the Ornithine Metabolic Pathway in Cancer Stem Cells

Jun Koseki^1,†^, Hidetoshi Matsui^2,†^, Masamitsu Konno^3,†^, Naohiro Nishida^4^, Koichi Kawamoto^3,4^, Yoshihiro Kano^3,4^, Masaki Mori^4,*^, Yuichiro Doki^4,*^, Hideshi Ishii^1,3,*^

^1^Department of Cancer Profiling Discovery, Graduate School of Medicine, Osaka University, Osaka 565-0871, Japan

^2^Faculty of Mathematics, Kyushu University, Fukuoka, 819-0395, Japan

^3^Department of Frontier Science for Cancer and Chemotherapy, Graduate School of Medicine, Osaka University, Osaka 565-0871, Japan

^4^Department of Gastroenterological Surgery, Graduate School of Medicine, Osaka University, Osaka 565-0871, Japan

**Supplementary Figure**

**
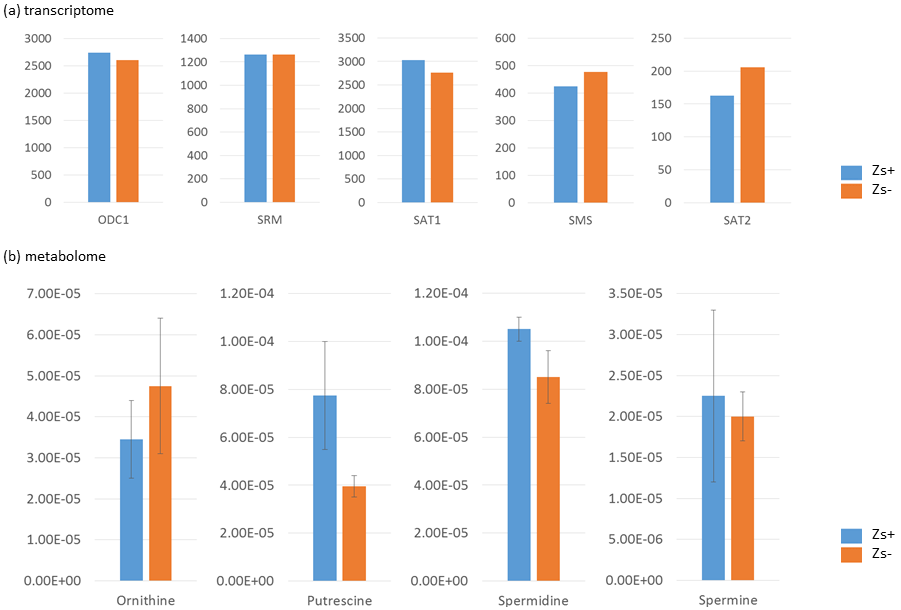
**

**Supplementary Figure 1.** The amounts of transcription and metabolism of Zs+ and Zs− cell at the zero time point (T_0_), involved in the polyamine metabolism pathway.

**
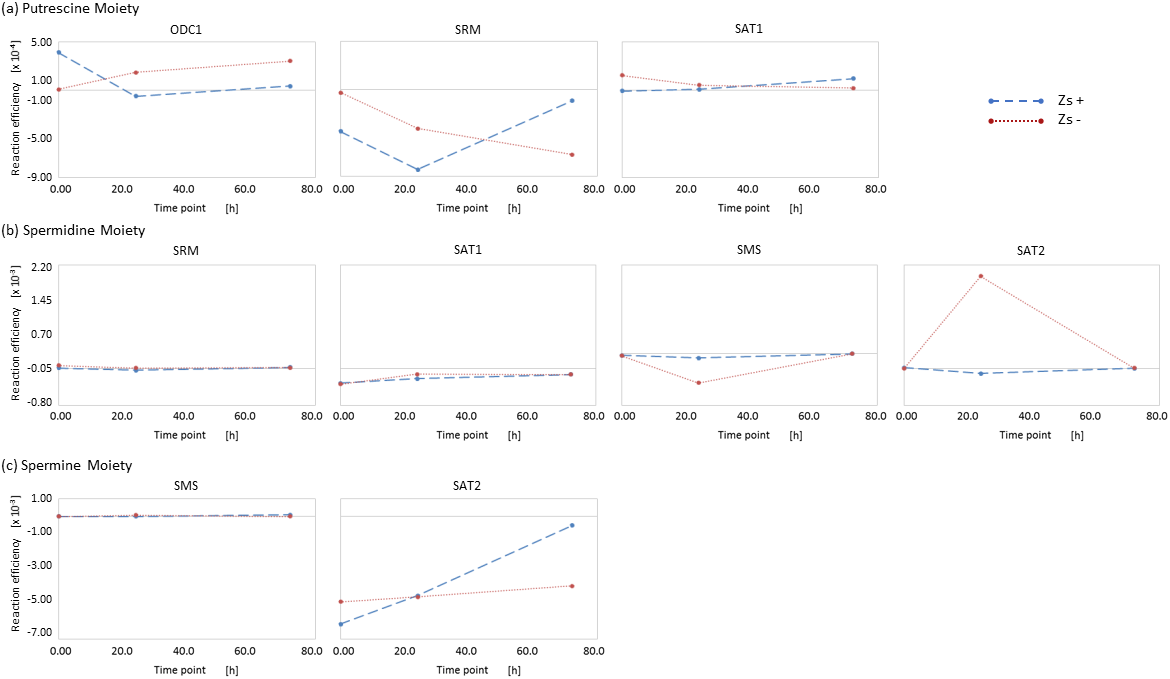
**

**Supplementary Figure 2.** The change over time of the coefficients of variability after exposure of Zs+ and Zs− cells to anti-tumour agents in each same order axis.
